# Relationship Between Ethnic Essentialism and Psychological Compatibility: Roles of Ethnic Identity and Self-Construal

**DOI:** 10.3390/bs14121207

**Published:** 2024-12-16

**Authors:** Jiani He, Yufang Zhao, Chao Zhang, Weichao Wang, Jinhua Huang

**Affiliations:** 1 Centre for Studies of Education and Psychology of Ethnic Minorities in Southwest China, Southwest University, Chongqing 400715, China; jianihepsy@163.com (J.H.); notfunnyjin@outlook.com (J.H.); 2 Student Mental Health Education and Service Centre, Student Affairs Department, Dali University, Dali 671003, China; 3 Faculty of Psychology, Southwest University, Chongqing 400715, China; chao0405@email.swu.edu.cn; 4 Anti-Narcotics Academy, Yunnan Police College, Kunming 650221, China; weichao_dali@163.com

**Keywords:** lay people’s theory, essentialist theory of ethnicity, social identity, independent, interdependent, intergroup relations

## Abstract

We examined the impact of ethnic essentialism on psychological compatibility among minority and Han Chinese college students and investigated the roles of ethnic identity and self-construal. A moderated mediator analysis was used and a multigroup comparison of the moderated mediator model across ethnic groups was conducted. The results indicate that ethnic essentialism significantly and negatively predicts psychological compatibility. Ethnic identity acts as a competitive mediator between ethnic essentialism and psychological compatibility, partially weakening the negative predictive effect of ethnic essentialism on psychological compatibility. Higher levels of ethnic identity lessen the negative impact of ethnic essentialism on psychological compatibility. Individuals with high independent and interdependent self-construal show higher levels of ethnic identity when their ethnic essentialism is strong. Under conditions of high ethnic identity, both high independent and high interdependent self-construal individuals exhibit significantly higher levels of psychological compatibility. In addition, ethnic differences were found in the moderated mediation model regardless of whether the moderating variable was independent or interdependent self-construal. These findings suggest that stronger beliefs in ethnic essentialism are associated with lower levels of psychological compatibility.

## 1. Introduction

Psychological compatibility, as a representation of positive intergroup relations, refers to psychological readiness for mutual acceptance and willingness to coexist harmoniously between individuals or groups [1]. The promotion of ethnic psychological compatibility is a key task used to strengthen the sense of community in China [2,3,4]. Empirical evidence suggests that factors such as social categorization, implicit theories of ethnicity, ethnic identity, ethnic stereotypes, prejudice, and intergroup threat can influence psychological compatibility among ethnic groups [4]. Ethnic essentialism, as an implicit theory for understanding group attributes (such as race, religion, and gender), affects how individuals process, judge, and respond to group information [5], influencing attitudes and preferences toward in- and outgroups [6,7].

With essentialist intuitions about social categories, viewing them as reflecting people’s intrinsic essences or biological natures, this intuition promotes prejudice development [8]. Therefore, higher levels of ethnic essentialism may lead to lower levels of psychological compatibility. An essentialist understanding of a group implies not only that group membership is immutable, but also that members share an underlying and identity-determining essence or nature [9]. Essentialist beliefs have been studied in relation to a wide range of phenomena, such as race [10], ethnicity [11], and personality [12]. However, in some instances, essentialism can be a strategy for reducing blame over individuals and groups and for identity formation [13]. Essentialism sometimes promotes prejudice, and other times mitigates it, influenced by various variables [8]. On the other hand, ethnic identity is a psychological pathway that influences ethnic integration [4], which can have both positive and negative effects on intergroup relations [14,15]. Self-construal refers to the process by which individuals define the self in relation to others, which underpins cognition, motivation, and social behavior [16]. Independence and interdependence exist in all cultures, and individuals hold both independent and interdependent aspects [17]. One of the questions the present study seeks to explore is whether self-construal is a factor influencing essentialism and ethnic identity. Additionally, a study of Chinese university students found that some people’s understanding of the nature of the nation tended to favor the national essentialist view [18]. In conclusion, the purpose of this study is to explore whether the role of ethnic essentialism on psychological compatibility in China changes under the influence of ethnic identity and self-construal, in order to provide a better understanding of the relationships among ethnic groups within the Chinese cultural context.

## 2. Literature Review

### 2.1. Relationship Between Ethnic Essentialism and Psychological Compatibility

Psychological essentialism, as a way of representing social categories [19], is the belief that entities have an “intrinsic essence” that gives rise to their observable characteristics [20], which could increase intergroup distance and hinder intergroup integration [21]. Individuals with essentialist beliefs think that social categories reflect the inherent, immutable characteristics of groups, believing that external, superficial differences are inevitably linked to underlying essential characteristics [19,22,23]. Individuals higher in essentialism were less likely to seek intergroup contact [24], while lower essentialist beliefs had greater outgroup empathy, which predicted more positive attitudes and pro-social behaviors across group lines [25]. Consequently, psychological essentialism significantly impacts intergroup relations [26], including among inter-ethnic groups [27,28]. Ethnic essentialists, compared with constructivists, exhibit stronger ethnic prejudices [29], more negative attitudes toward outgroups [6], and impediments to cross-ethnic interactions [19]. The impact of essentialism on intergroup attitudes has been confirmed across cultures. A study of Jewish children, when exposed to racial essentialism, depicted greater distances between Jews and Arabs, exhibiting stronger prejudice against Arabs [30]. A study in China revealed that the Tibetan students who initiated ethnic essentialism in the experiment demonstrated a reduced willingness to engage in inter-ethnic interactions with Han Chinese [19].

Essentialists, who view social identities as fixed, also view these identities as capable of guiding self-conception and intergroup orientation [9]. Individuals prefer to align with groups that are high in entitativity or high in inductive potential, which are the components of social essentialism [31]. Identification with a social category may thus rely on the category being somewhat essentialized [13]. Bastian and Haslam [9] found that essentialist beliefs among immigrants moderated their adoption of identity as a self-guide during acculturation. Furthermore, social identity processes come into play when people categorize themselves and others as group members [32]. Social identity theory suggests that people form their individual identities through their identification with social categories [33,34]. The categorization might also entail intergroup biases or intergroup discrimination in favor of the ingroup [35]. The degree of intergroup psychological compatibility can be an indicator of intergroup relations [36]. Psychological compatibility reflects the strengths and weaknesses of the group relationship. A higher degree of psychological compatibility indicates that the individual is more willing to accept the outgroup, has a higher appraisal of the outgroup, and is more willing to help others and interact with them [36]. It is thus hypothesized that psychological compatibility is negatively related to ethnic essentialism (H1).

### 2.2. Role of Ethnic Identity Between Ethnic Essentialism and Psychological Compatibility

Ethnic identity is a fundamental variable for measuring ethnic interactions and relations [4]. It reflects ethnic members’ affirmation and emotional attachment to their ethnic identity [37,38]. Ethnic identity may also be a mechanism of action that predicts the relationship between psychological essentialism and psychological compatibility, as it refers to the degree of identification with one’s culture, activities, history, and language based on ethnic identity [39]. Psychological essentialism has been demonstrated to influence individuals’ group identity processes [9]; the stronger the belief in ethnic essentialism, the higher the level of ingroup identity [14]. Furthermore, ethnic identity influences individuals’ perceptions and behaviors regarding ethnic relations. A heightened level of awareness of ethnic groups and ethnic relations, which can be defined as a mature ethnic identity [40], is associated with a more positive level of inter-ethnic attitudes [41]. Research on minority college students in China found that ethnic identity is significantly positively correlated with attitudes toward outgroup interactions [14]. As the level of psychological compatibility is an index of the quality of intergroup attitudes and the closeness of group distances [3], it can be inferred that ethnic identity predicts psychological compatibility. Based on research indicating that ethnic identity is related to both psychological compatibility and ethnic essentialism, we hypothesize that ethnic essentialism predicts psychological compatibility through ethnic identity (H2).

### 2.3. Moderating Role of Self-Construal

Self-construal is a manifestation of cultural orientation in constellations of thought, feeling, and behavior, which are directly related to the self [42,43]. Current research suggests that self-construal may be closely related to both ethnic essentialism and ethnic identity. First, essentialism is believed to set each person apart from others, be naturally occurring, and be temporally and situationally consistent [44], which may be related to how individuals view themselves in cultural contexts [45,46,47,48]. A major site of cultural influence in views of the self is the value each culture places on individualistic (e.g., individual-level) and collectivistic (e.g., group-level) goals [49]. Individualists and collectivists differ in the extent to which they view the self as independent or interdependent of their social group [50]. Due to the West’s emphasis on individual-level goals and each person’s unique aspects, one might expect Westerners to be more likely to engage in psychological essentialism compared with people of other cultural backgrounds [49]. For example, Americans show a positive relationship between individualism and self-essentialism [44,50], while the Japanese exhibit slight self-essentialism endorsement as well, albeit significantly lower in magnitude than their American counterparts [50]. Among Tibetan Buddhist Monks, interdependent self-construal moderated the association of self-essentialism with satisfaction with life [49]. The implications of the above studies are that self-construal predicts psychological essentialism differently in various cultural contexts. In summary, we hypothesize that the impact of ethnic essentialism on ethnic identity is moderated by self-construal (H3).

**Figure 1:**
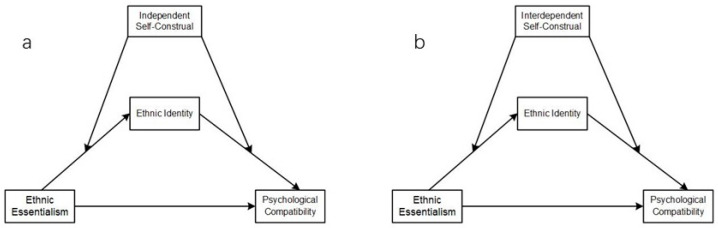
Hypothetical model of the mediating role of ethnic identity and the moderating roles of (**a**) independent and (**b**) interdependent self-construal.

Furthermore, ethnic identity is closely related to self-construal [51]. Differences in cultural orientation cause differences in self-construal, which influence identity formation; thus, interdependent self-construal could mediate the role of cultural orientation in identity formation [52]. Self-expression and identity are malleable and have been theorized to be influenced by context, particularly for individuals from collectivistic cultures [53]. Collectivist values that are associated with an interdependent self-construal are also associated with higher identification with the ingroup [54,55], finding that a person may possess components of both an individualist and a collectivist personality. Considering the role of cultural differences in Chinese culture, self-construal may have implications for ethnic essentialism and ethnic identity. A study of different ethnic groups in China found that Tibetans have higher levels of collectivism than Han Chinese [56]. Accordingly, self-construal moderates the relationship between ethnic identity and psychological compatibility (H4) (Figure 1).

### 2.4. Ethnic Differences in the Moderated Mediation Model

As a unified country with 56 ethnic groups, China has a rich and colorful ethnic culture [57], and there are many differences in the psychological characteristics of different ethnic groups [58,59]. The Han Chinese are the majority ethnic group in China. In terms of cognition, the white color is often associated with light and justice in the Han culture, while black symbolizes evil [60]. The Yi people refer to themselves as “black” people and their traditional attire is predominantly black [61]. Studies have found that cognitive reappraisal is more effective in regulating negative emotions in Uyghur individuals compared with Han Chinese [62], and Han college students are more inclined to use cognitive reappraisal strategies to regulate negative emotions than Tibetan college students [63]. There are also differences in the human dimension between the majority ethnic and minorities. For example, the Naxi are more active, proactive, and positive in interpersonal situations than the Han Chinese; they are more rigorous and self-controlled in their individual ways and attitudes [64]. In addition, subjective well-being research has found that Uyghurs and Hui have significantly higher well-being than Han Chinese, and the level of well-being of Zhuang individuals is slightly lower than that of Han Chinese [65]. Based on the discrepancies between the Chinese majority and minority ethnic groups, we proposed that the relationship model of ethnic essentialism, psychological compatibility, ethnic identity, and self-construal differs between the majority ethnic group and ethnic minorities (H5).

### 2.5. The Present Study

Many studies have demonstrated the inhibiting effect of essentialism on intergroup relations, but the role of ethnic identity in ethnic relations has both positive and negative effects [14,66]. Based on this, the present study further explored the role of ethnic identity in intergroup relations. Guided by the essentialist theory of ethnicity and social identity, we proposed a moderated mediation model to investigate the mechanisms underlying the association between ethnic essentialism and psychological compatibility. The moderating and mediating mechanisms have yet to be examined. We assumed that the relationship between ethnic essentialism and psychological compatibility is negatively related (H1). Moreover, ethnic identity would moderate the relationship between ethnic essentialism and psychological compatibility, such that people with higher ethnic essentialism would report more psychological compatibility when their ethnic identity is high (H2). Furthermore, we predicted that self-construal mediates the moderating effect of ethnic essentialism and ethnic identity; that is, the interactions between ethnic essentialism and ethnic identity, and ethnic identity and psychological compatibility, in a model of psychological compatibility go through a mediator (H3 and H4).

In addition, there are significant differences in areas, such as living conditions and cultural practices, between the various ethnic groups in China [67]; we explored possible ethnic differences within this moderated mediation model (H5).

## 3. Materials and Methods

### 3.1. Participants

In this study, questionnaires were administered in three universities in Yunnan Province and Chongqing Municipality, China. The link to complete the survey online was distributed in class by the instructor, and all participants volunteered to take this survey. Moreover, each subject read the informed consent form before filling out the questionnaire, and those who satisfactorily completed it received compensation.

A total of 989 questionnaires were distributed via a survey website www.wenjuan.com (accessed on 26 April 2023), and 952 valid responses were retained after excluding those who failed two attention-check questions (e.g., “Please select ‘somewhat agree’”) or had completion times that were too short or too long. The final sample consisted of 433 men and 519 women, aged 18–33 years (*M* = 21.53, *SD* = 2.20). The sample consisted of 608 participants from the ethnic majority participants, that is, Han Chinese, and 344 participants from ethnic minorities.

### 3.2. Research Instruments

#### 3.2.1. Ethnic Essentialism Questionnaire

The Lay Theory of Race Scale developed by No and Hong [68] was used. In Wan and Gao’s study [14], this scale was translated and revised, and it was applied to ethnic groups in China. The scale comprises eight items. The first four items assess the degree to which participants hold essentialist views about ethnicity, with a typical item being “It is hard if not impossible to change the depositions of a person’s race”. The latter four items assess constructivist views, with a typical item being “Races are just arbitrary categories and can be changed if necessary”. The item is rated using a 6-point Likert scale (1 = strongly disagree, 2 = disagree, 3 = somewhat disagree, 4 = slightly agree, 5 = agree, 6 = strongly agree). The latter four items are reverse-scored before calculating the total score. Higher total scores indicate stronger essentialist views, whereas lower scores indicate stronger constructivist views. In this study, the coefficient α for the ethnic essentialism and ethnic constructivism subscales was 0.872 and 0.813, respectively, with an overall scale coefficient α of 0.667.

#### 3.2.2. Psychological Compatibility Measurement

Psychological distance was used as an indicator of psychological compatibility, measured by the Inclusion of Other in the Self Scale [69,70]. This scale consists of one item and is presented in pictorial form, consisting of two circles, and relationship closeness is indicated by varying the degree of distance between the intersection of the two circles. The item is rated using a 7-point Likert scale, with higher scores indicating a higher degree of psychological compatibility with the target group.

#### 3.2.3. Ethnic Identity Questionnaire

The Ethnic Affirmation subscale, consisting of six items from the Ethnic Identity Scale [71], was used. A typical item is “I am happy to be a member of my ethnic group”. Higher scores indicate a stronger affirmation of one’s ethnic identity and a stronger sense of belonging to one’s ethnic group. In this study, the internal consistency coefficient *α* for the scale was 0.861.

#### 3.2.4. Self-Construal Scale

The Chinese version of the Self-Construal Scale [72] was used, which consists of 24 items, with 12 items each for independent and interdependent self-construal. A typical item for independent self-construal is “Speaking up in class (or meetings) is not a problem for me”. A typical item for interdependent self-construal is “I will stay with the group if needed, even if I am not happy being with them”. This instrument uses a 7-point scale (1 = strongly disagree, 7 = strongly agree). Higher scores indicate higher levels of independent and interdependent self-construal. In this study, the internal consistency coefficients αs for the independent and interdependent Self-Construal Scales were 0.844 and 0.868, respectively.

### 3.3. Statistical Analysis

All data were checked for missing values before analysis. Missing values were replaced by the means of the measures because the mean may be the best estimate of the value of a variable in the absence of all other information, and this procedure is conservative as it does not change the mean of the distribution as a whole [73]. We performed common method bias tests, descriptive statistics, correlation analysis, and tests of differences in main variables using SPSS 25.0, moderated mediation analysis using SPSS macro-PROCESS v4.2, and a multigroup comparison of the moderated mediation model using both AMOS 24.0 and the Stats Tools Package. As noted above, four hypotheses formed a moderated mediation model. A moderated mediation relationship is said to occur when a mediation relationship depends upon the level of a moderator [74]. Thus, the data analysis was performed according to the following four steps. First, as all data were collected using self-report scales, we conducted an assessment for common method bias (CMB), a type of measurement error that can threaten the validity of conclusions about relationships between measures and influence the results of behavioral research [75]. Second, the mediating role of ethnic identity was tested using SPSS macro-PROCESS Model 4 [76], and we examined the mediating effect of ethnic identity on the relationship between ethnic essentialism and psychological compatibility, while controlling for ethnicity, age, and gender. Third, we tested the influence of independent and interdependent self-construal as the moderating variable in the model. Bias-corrected bootstrap confidence intervals (CIs), based on 5000 bootstraps [77,78], were calculated to test the significance of the mediating effect. Fourth, we conducted a multigroup analysis to test for ethnic differences in the proposed moderated mediation model.

### 3.4. Ethical Considerations

This study was approved by the Faculty of Psychology, Southwest University (IRB No.: H23120). All participants provided informed consent.

## 4. Results

### 4.1. Control and Testing of Common Method Bias

Using a questionnaire method can lead to common method bias. To mitigate this, the anonymity of respondents was protected, and some items were reverse-scored [79]. Additionally, Harman’s single-factor test was used to assess common method bias [77]. The results showed that there were six factors with eigenvalues greater than 1, and the variance explained by the first factor was 30.77%, which is less than the 40% threshold. Therefore, the data in this study do not exhibit severe common method bias.

### 4.2. Descriptive Statistics and Correlation Analysis of Variables

Descriptive statistics and correlation analysis of the main variables are shown in Table 1. Ethnic essentialism was significantly and negatively correlated with psychological compatibility and independent self-construal, and significantly and positively correlated with ethnic identity. However, it was not significantly correlated with interdependent self-construal. Psychological compatibility was significantly and positively correlated with ethnic identity, independent self-construal, and interdependent self-construal. Ethnic identity was significantly and positively correlated with both independent and interdependent self-construal.

### 4.3. Test of Differences in Main Variables Between Ethnic Majority and Minorities

An independent-samples *t*-test was used to compare differences in ethnic essentialism, ethnic identity, psychological compatibility, and self-construal between the majority and minority ethnic groups. It was found that there was a significant difference between the majority and minority ethnic groups in terms of ethnic identity (*t* = 3.261, *p* < 0.001) and independent self-construal (*t* = 3.261, *p* < 0.05), but no significant difference in any of the other variables (Table 2).

### 4.4. Mediation Effect Analysis

The analysis showed that, after controlling for ethnicity, age, and gender, ethnic essentialism significantly and negatively predicted psychological compatibility (*B* = −0.173, *t* = −2.188, *p* < 0.05), confirming H1. When the mediator variable was included, the direct negative predictive effect of ethnic essentialism on psychological compatibility remained significant (*B* = −0.349, *t* = −4.706, *p* < 0.001), and it significantly and positively predicted ethnic identity (*B* = 0.215, *t* = 5.739, *p* < 0.001). Additionally, ethnic identity significantly and positively predicted psychological compatibility (*B* = 0.818, *t* = 12.950, *p* < 0.001). The bias-corrected percentile bootstrap method indicated that the mediating effect of ethnic identity between ethnic essentialism and psychological compatibility was significant, a × b = 0.176, *SE* = 0.035, 95% bootstrap *CI* [0.046, 0.100]. Furthermore, the direct and indirect effects of ethnic essentialism on psychological compatibility were in opposite directions, indicating a competitive mediating effect of ethnic identity [76]. The mediating effect accounted for 33.54% of the total effect, confirming H2 (Table 3).

### 4.5. Moderated Mediation Analysis of Self-Construal

Consistent with the theoretical model, we tested the moderated mediation model after controlling for ethnicity, age, and gender using Model 58 from the SPSS macro program [77].

**Figure 2:**
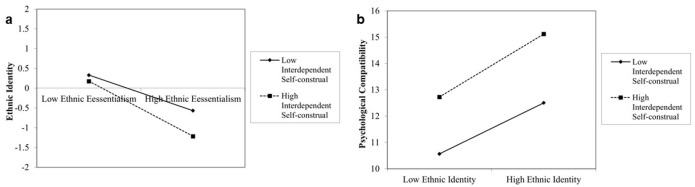
(**a**) Simple slopes of independent self-construal moderating the relationship between ethnic essentialism and ethnic identity and (**b**) between ethnic identity and psychological compatibility.

**Figure 3:**
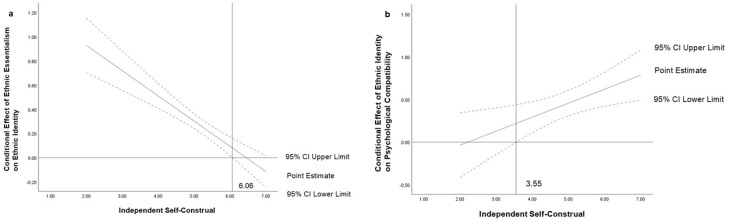
(**a**) Johnson-Neyman plots of independent self-construal moderating the relationship between ethnic essentialism and ethnic identity and (**b**) between ethnic identity and psychological compatibility.

First, we included independent self-construal as the moderating variable in the model. The results (Table 4) indicated that the interaction term between ethnic essentialism and independent self-construal significantly and negatively predicted ethnic identity (*B* = −0.208, *t* = −6.076, *p* < 0.001). Additionally, the interaction term between ethnic identity and independent self-construal significantly and positively predicted psychological compatibility (*B* = 0.164, *t* = 2.664, *p* < 0.01). This suggests that independent self-construal moderates not only the prediction of ethnic identity by ethnic essentialism but also the prediction of psychological compatibility by ethnic identity. Further simple slope analysis [80] showed that for participants with lower levels of independent self-construal (*M − 1 SD*), the negative predictive effect of ethnic essentialism on ethnic identity was significant (*t* = −4.253, *p* < 0.001) (Figure 2a). For participants with higher levels of independent self-construal (*M + 1 SD*), the negative predictive effect of ethnic essentialism on ethnic identity was significant and stronger (*t* = −4.763, *p* < 0.001). For participants with lower levels of independent self-construal (*M − 1 SD*), the positive predictive effect of ethnic identity on psychological compatibility was significant (*t* = 4.038, *p* < 0.001) (Figure 2b). For participants with higher levels of independent self-construal (*M + 1 SD*), the positive predictive effect of ethnic identity on psychological compatibility was significant and stronger (*t* = 3.700, *p* < 0.001). Using the Johnson–Neyman technique for simple slope tests [81], Figure 3a shows that when independent self-construal ≤ 6.06, the 95% confidence interval is above 0, indicating a significant effect of ethnic essentialism on ethnic identity. When independent self-construal > 6.06, the 95% confidence interval includes 0, indicating no significant effect. Figure 3b shows that when independent self-construal ≥ 3.55, the 95% confidence interval is above 0, indicating a significant effect of ethnic identity on psychological compatibility. When independent self-construal < 3.55, the 95% confidence interval includes 0, indicating no significant effect.

**Figure 4:**
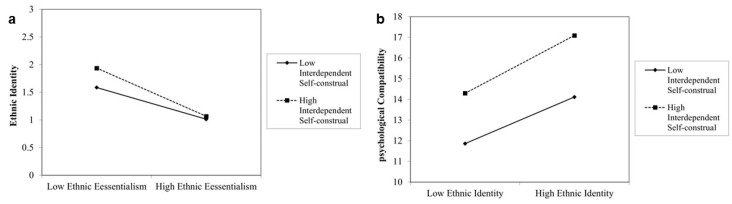
(**a**) Simple slopes of interdependent self-construal moderating the relationship between ethnic essentialism and ethnic identity and (**b**) between ethnic identity and psychological compatibility.

**Figure 5:**
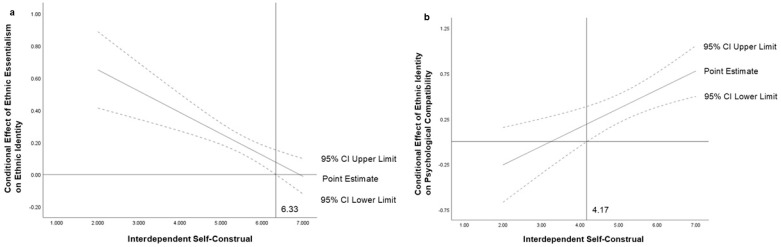
(**a**) Johnson-Neyman plots of interdependent self-construal moderating the relationship between ethnic essentialism and ethnic identity and (**b**) between ethnic identity and psychological compatibility.

Next, interdependent self-construal was included as the moderating variable in the model. The results (Table 5) indicated that the interaction term between ethnic essentialism and interdependent self-construal significantly negatively predicted ethnic identity (*B* = −0.132, *t* = −3.989, *p* < 0.001). Additionally, the interaction term between ethnic identity and interdependent self-construal significantly positively predicted psychological compatibility (*B* = 0.206, *t* = 3.309, *p* < 0.01). This suggests that interdependent self-construal also moderates the prediction of ethnic identity by ethnic essentialism and the prediction of psychological compatibility by ethnic identity. For participants with lower levels of interdependent self-construal (*M* − *1 SD*), the negative predictive effect of ethnic essentialism on ethnic identity was significant (*t* = −2.633, *p* < 0.01) (Figure 4a). For participants with higher levels of interdependent self-construal (*M + 1 SD*), the negative predictive effect of ethnic essentialism on ethnic identity was significant and stronger (*t* = −2.986, *p* < 0.01). For participants with lower levels of interdependent self-construal (*M* − *1 SD*), the positive predictive effect of ethnic identity on psychological compatibility was significant (*t* = 4.411, *p* < 0.001) (Figure 4b). For participants with higher levels of interdependent self-construal (*M + 1 SD*), the positive predictive effect of ethnic identity on psychological compatibility was significant and stronger (*t* = 4.168, *p* < 0.001). Using the Johnson–Neyman technique for simple slope tests, Figure 5a shows that when interdependent self-construal ≤ 6.33, the 95% confidence interval is above 0, indicating a significant effect of ethnic essentialism on ethnic identity. When interdependent self-construal > 6.33, the 95% confidence interval includes 0, indicating no significant effect. Figure 5b shows that when interdependent self-construal ≥ 4.17, the 95% confidence interval is entirely above 0, indicating a significant effect of ethnic identity on psychological compatibility. When interdependent self-construal < 4.17, the 95% confidence interval includes 0, indicating no significant effect.

### 4.6. Multigroup Comparison of Moderated Mediation Model Between Ethnic Majority and Minorities

Multigroup models were created using AMOS 24.0, and the Stats Tools Package: http://www.gaskination.com/Stats%20Tools%20Package.xlsm (accessed on 10 February 2024) was employed to test the ethnic differences in the moderated mediation model. The results are presented in Table 6. When independent self-construal was used as a moderating variable, there were significant ethnic differences in the prediction of psychological compatibility by ethnic identity (*z* = 2.733, *p* < 0.01), with nonsignificant differences for the remaining pathways. When interdependent self-construal was used as a moderating variable, there were significant ethnic differences in the effects of ethnic essentialism (*z* = 18.995, *p* < 0.01), interdependent self-construal (*z* = 14.352, *p* < 0.01), and the interaction of ethnic essentialism and interdependent self-construal (*z* = −24.136, *p* < 0.01) on ethnic identity among both majority and minority ethnic groups. Moreover, significant ethnic differences in ethnic identity (*z* = 4.323, *p* < 0.01) and the interaction effect of ethnic identity and interdependent self-construal (*z* = −2.188, *p* < 0.05) on psychological compatibility, as well as a nonsignificant difference in the effect of ethnic essentialism on psychological compatibility, were found.

## 5. Discussion

We explored the relationship between ethnic essentialism and psychological compatibility, and how ethnic identity and self-construal affect this relationship. It was found that ethnic essentialism significantly and negatively predicted psychological compatibility. Ethnic identity acted as a competitive mediator between ethnic essentialism and psychological compatibility. Moreover, both pathways—the prediction of ethnic identity by ethnic essentialism and of psychological compatibility by ethnic identity—were moderated by high independent and interdependent self-construal.

Ethnic essentialism negatively and significantly predicting psychological compatibility is consistent with previous research [82,83,84], as ethnic essentialism makes the intergroup boundaries solid. The formation of essentialist beliefs about ethnicity serves to reinforce intergroup boundaries, perceived intergroup differences [14], and unwillingness to socialize with outgroups that differ from the ingroup. Additionally, group cognitive differences increase, stereotypes and prejudices about external groups are strengthened [22], and all of them are not conducive to cross-ethnic interaction and communication and mingling [20]. In summary, essentialism brings about difficulties in crossing ethnic boundaries, which is not conducive to good group relations and thus hinders group psychological compatibility.

Ethnic identity significantly reduces the negative predictive power of ethnic essentialism on psychological compatibility because it enhances epistemic security. According to social identity theory, people gain a positive social identity by belonging to a group, and when people ascribe essentialist beliefs to social groups, social identities are viewed as immutable entities and enhance the psychological effects of social identity [85]. The positive social identity reduces the sense of impermanence in social life, provides epistemic security, and helps people determine how to interact with others [86]. Stable ethnic identity can play a positive role in attitudes toward inter-ethnic contact [87]. Research in China showed that individuals from different ethnic groups exhibit a stronger ethnic identity [88,89], which suggests that ethnic groups in China are likely to have a mature and stable ethnic identity. Consequently, when ethnic essentialism reinforces ethnic identity, what is activated in the Chinese cultural context is a stable and mature ethnic identity, which may facilitate intergroup interactions and enhance psychological compatibility by increasing an individual’s sense of security in intergroup interactions.

The current study found that essentialism has generally been regarded as a harmful cognitive process that results in prejudice and other forms of injustice at the group level. However, in some instances, essentialism can be a strategy for reducing blame over individuals and groups and for identity formation. This is consistent with previous research [13]. Self-construal as a moderator played an important role in identity formation and intergroup relations. In the relationship between ethnic essentialism, ethnic identity, and psychological compatibility, compared with low independent and interdependent self-construal, those with high independent and interdependent self-construal showed a stronger positive prediction of ethnic identity through ethnic essentialism and a stronger positive prediction of psychological compatibility through ethnic identity. It is worth noting that the mode of action of high independent and interdependent self-construal is the same in the relationship between ethnic essentialism, ethnic identity, and psychological compatibility. In the context of globalization, collectivist societies increasingly encounter individual-oriented values, while relationship-oriented collective values are more likely to appear in Western individualistic societies [90,91]. In Chinese culture, a “bicultural self” has evolved to cope with and accompany social changes, and the bicultural self—that is, higher levels of both independent and interdependent self-construal—has a higher social adaptive capacity to cope with social and cultural changes [91,92]. In addition, the mechanism of high independent and interdependent self-construal may differ in the relationship between ethnic essentialism, ethnic identity, and psychological compatibility. Individuals with independent self-construal emphasize personal autonomy and independence, focus on the expression and affirmation of positive internal attributes, pursue self-actualization [93], and may gain group identity based on positing competence [94]. Individuals with a high level of interdependent self-construal may develop positive social relationships based on the need for interpersonal harmony [95,96]. Independent and interdependent self-construal correspond to “autonomy” and “interpersonal connections”, respectively [97,98]. As fundamental human requirements, they are not mutually exclusive but should be mutually reinforcing in terms of long-term individual development outcomes [99].

In addition, we found an ethnic difference within the moderated mediation model, as expected. Only significant ethnic differences existed in the pathway of positively predicting psychological compatibility despite ethnic identity, when independent self-construal was used as a moderating variable. Meanwhile, when interdependent self-construal was employed as a moderating variable, except for the path of the effect of ethnic essentialism on psychological compatibility, where there were no significant ethnic differences, there were significant ethnic differences in all other paths.

The discrepancies between majority and minority communities may be attributed to dissimilarities in a multitude of domains, which may impact each individual belonging to a different ethnic group. According to the developmentally oriented view of ethnic identity, a mature ethnic identity implies a higher level of awareness of ethnic groups and the relationships among them and may also imply a more positive understanding of intergroup relations [40]. The extent of belonging to an ethnic group is shaped by the corresponding ethnic culture. For example, Tibetans form closer interconnections and exhibit higher levels of collectivism than individuals in Han cultural areas to ensure their survival on the Tibetan Plateau, where geographic and climatic conditions are relatively harsh [56]. Therefore, the cultural context, as a repository of collective thoughts, feelings, and values, exerts a significant influence on the cognitive style of individual fields [100]. In summary, the differences between the majority and minority ethnic groups regarding ethnic identity and culture may be attributed to the ethnic differences observed in this study.

One strength of the current study lies in the fact that the effects of essentialism on intergroup relations do not necessarily lead only to negative outcomes. In other words, essentialism may be a flexible conversational resource [101,102] and a strategy that contributes to the formation of personal identity [13]. Mixed-type self-construal, which is the people who assign the same degree of value to both independent and interdependent self-construal [103], is a factor moderating essentialism. Furthermore, in Chinese culture, strengthening ethnic identity mitigating the negative impact of ethnic essentialism on psychological compatibility provides new evidence for understanding ethnic cognition and ethnic relations. Although Chinese people currently tend to hold essentialist views on ethnicity [18], the relationships among the 56 ethnic groups are characterized by mutual assistance and shared destiny, akin to pomegranate seeds tightly embracing each other [104]. Additionally, high independent and interdependent self-construal simultaneously moderate ethnic essentialism and ethnic identity, enriching the understanding of the diversity and dynamism of self-construal theory. Independent and interdependent self-construal may exhibit similar patterns in certain psychological processes. Under the influence of globalization, with extensive and in-depth exchanges and interactions among different cultures, further research is needed on the role of self-construal in individual and group psychological processes and behaviors.

In this study, ethnic minorities represented a collection of multiple ethnic groups rather than a single ethnicity, which might mask potential differences among ethnic groups because of the differences among ethnic minorities in the Chinese culture [67]. In addition, most of the ethnic minority participants in this study were from southwest China, and the ethnic minorities in northwest and southwest China may have been different. Future research needs to focus on the similarities and differences among ethnic minorities. Furthermore, this was a correlational study using measurements and could not reveal causality; the results should be verified by using experimental methods in the future.

## 6. Conclusions

Using a sample of ethnic groups in China, this study explored the relationship between ethnic essentialism and psychological compatibility and its underlying mechanisms. The findings indicate that ethnic essentialism is a significant negative predictor of psychological compatibility. Ethnic identity undermines the negative prediction of psychological compatibility by ethnic essentialism, and high independence and interdependence self-construal acted as moderators of ethnic essentialism and ethnic identity. Furthermore, ethnic differences were found in the moderated mediation model, regardless of whether the moderating variable was independent or interdependent self-construal. The findings highlighted that in some instances, under the influence of certain factors, essentialism may not necessarily be a harmful cognitive process but may be a strategy for reducing blame over individuals and groups and for identity formation. Self-construal as a moderator played an important role in identity formation and intergroup relations. Thus, this study not only provides a new theoretical perspective on the relationship between ethnic essentialism and psychological compatibility, but also reveals the potential mechanism of the role of self-construal in this relationship.

## Figures and Tables

**Table 1:** Descriptive statistics and correlation analysis of variables.

Variables	*M*	*SD*	1	2	3	4
1. Ethnic Essentialism	3.735	0.685				
2. Psychological Compatibility	4.430	1.677	−0.087 **	-		
3. Ethnic Identity	4.932	0.799	0.178 ***	0.370 ***	-	
4. Independent Self-Construal	5.234	0.865	−0.071 *	0.442 ***	0.567 ***	-
5. Interdependent Self-Construal	5.444	0.816	0.028	0.443 ***	0.640 ***	-

* p < 0.05, ** *p* < 0.01, *** *p* < 0.001.

**Table 2:** Tests of differences between the ethnic majority and minorities on main variables.

	Ethnic Majority		Ethnic Minorities				Cohen’s *d*
Variables	*M*	*SD*	*M*	*SD*	*t*	*p*	
Ethnic Essentialism	3.745	0.678	3.717	0.697	0.610	0.542	0.041
Psychological Compatibility	4.488	1.662	4.334	1.701	1.364	0.173	0.092
Ethnic Identity	4.996	0.774	4.821	0.830	3.261	0.001	0.220
Independent Self-Construal	5.283	0.847	5.146	0.892	2.361	0.018	0.159
Interdependent Self-Construal	5.470	0.817	5.399	0.813	1.279	0.201	0.086

**Table 3:** Mediation model test of ethnic identity between ethnic essentialism and psychological compatibility.

Variables	Psychological Compatibility			Ethnic Identity			Psychological Compatibility		
	*B*	*SE*	*p*	*B*	*SE*	*p*	*B*	*SE*	*p*
Ethnicity	0.027	0.013	<0.05	−0.002	0.006	0.7	0.029	0.012	<0.05
Age	0.108	0.026	<0.001	0.028	0.012	<0.05	0.084	0.024	<0.001
Gender	−0.301	0.113	<0.01	−0.076	0.054	0.155	−0.239	0.104	<0.05
Ethnic Essentialism	−0.173	0.079	<0.05	0.215	0.038	<0.001	−0.349	0.074	<0.001
Ethnic Identity							0.818	0.063	<0.001
*R* ^2^	0.03			0.039			0.175		
*F* (*df*)	7.382 ***			15.402 ***			40.485 ***		

*** *p* < 0.001.

**Table 4:** Moderated mediation model test with independent self-construal as the moderating variable.

Variables	Ethnic Identity			Psychological Compatibility		
	*B*	*SE*	*p*	*B*	*SE*	*p*
Ethnicity	−0.001	0.005	0.856	0.025	0.012	<0.05
Age	−0.001	0.01	0.959	0.059	0.023	<0.01
Gender	0.098	0.043	<0.05	−0.078	0.102	0.442
Ethnic Essentialism	0.253	0.03	<0.001	−0.22	0.073	<0.001
Independent Self-Construal	0.545	0.024	<0.001	0.57	0.07	<0.001
Ethnic Essentialism × Independent Self-Construal	−0.208	0.034	<0.001			
Ethnic Identity				0.495	0.078	<0.001
Ethnic Identity × Independent Self-Construal				0.164	0.062	<0.01
*R* ^2^	0.396			0.241		
*F* (*df*)	103.332 ***			42.785 ***		

*** *p* < 0.001.

**Table 5:** Moderated mediation model test with interdependent self-construal as the moderating variable.

Variables	Ethnic Identity			Psychological Compatibility		
	*B*	*SE*	*p*	*B*	*SE*	*p*
Ethnicity	−0.006	0.005	0.234	0.023	0.012	0.055
Age	−0.001	0.009	0.933	0.059	0.023	<0.01
Gender	0.058	0.041	0.161	−0.118	0.101	0.242
Ethnic Essentialism	0.194	0.029	<0.001	−0.281	0.072	<0.001
Interdependent Self-Construal	0.618	0.024	<0.001	0.639	0.077	<0.001
Ethnic Essentialism × Interdependent Self-Construal	−0.132	0.033	<0.001			
Ethnic Identity				0.455	0.082	<0.001
Ethnic Identity × Interdependent Self-Construal				0.206	0.062	<0.01
*R* ^2^	0.446			0.243		
*F* (*df*)	127.009 ***			43.176 ***		

*** *p* < 0.001.

**Table 6:** Comparison of path coefficient differences between the ethnic majority and minorities in the moderated mediation model.

Mediation Path			Ethnic Majority		Ethnic Minorities		z-Score
			*B*	*p*	*B*	*p*	
Ethnic Essentialism	→	Ethnic Identity	1.35	0.000	1.274	0.000	−1.221
Ethnic Essentialism × Independent Self-Construal	→	Ethnic Identity	−0.207	0.000	−0.197	0.000	1.158
Independent Self-Construal	→	Ethnic Identity	1.298	0.000	1.286	0.000	−0.257
Ethnic Identity	→	Psychological Compatibility	−0.235	0.000	−0.066	0.000	2.733 ***
Ethnic Essentialism	→	Psychological Compatibility	−0.291	0.004	−0.145	0.273	0.882
Ethnic Identity × Independent Self-Construal	→	Psychological Compatibility	0.129	0.000	0.112	0.000	−1.289
Ethnic Essentialism	→	Ethnic Identity	0.492	0.000	1.624	0.000	18.995 ***
Ethnic Essentialism × Interdependent Self-Construal	→	Ethnic Identity	−0.057	0.000	−0.254	0.000	−24.136 ***
Interdependent Self-Construal	→	Ethnic Identity	0.834	0.000	1.56	0.000	14.352 ***
Ethnic Identity	→	Psychological Compatibility	−0.492	0.000	−0.163	0.000	4.323 ***
Ethnic Essentialism	→	Psychological Compatibility	−0.372	0.000	−0.186	0.167	1.136
Ethnic Identity × Interdependent Self-Construal	→	Psychological Compatibility	0.152	0.000	0.123	0.000	−2.188 **

** *p* < 0.01, *** *p* < 0.001.

## Data Availability

The data that support the findings of this study are available from the corresponding author, upon reasonable request.
